# Hybrid intervention with angio-CT for percutaneous transhepatic biliary drainage (PTBD)

**DOI:** 10.1007/s00261-025-05337-5

**Published:** 2025-12-12

**Authors:** Jonathan Nadjiri, Tobias Geith, Tobias Waggershauser, Marc Mühlmann, Philipp Paprottka

**Affiliations:** Ismaninger Str 22, TUM Klinikum Munchen, TUM School of Medicine and Health, Interventionelle Radiologie, München, Germany

**Keywords:** PTBD, Hybrid angio-CT, Aerobilia, Biliary intervention, Fluoroscopy, Multimodal imaging

## Abstract

**Purpose:**

To evaluate a hybrid angiography-CT (angio-CT) system for percutaneous transhepatic biliary drainage (PTBD) in patients with biliodigestive anastomosis, aerobilia, and the need for external biliary drainage.

**Materials and methods:**

Ten patients with bile duct leakage and aerobilia underwent angio-CT-guided PTBD. The angio-CT enabled combined CT and fluoroscopic imaging in a single procedural setting. Aerobilia facilitated bile duct visualization, particularly in left-sided ducts. Key steps included CT-guided puncture, fluoroscopic catheter placement, and integration of both modalities to streamline workflow.

**Results:**

Successful biliary access and drainage were achieved in all cases. The combined imaging approach allowed for short intervention times (mean: 25 min) and ensured safety without complications. Aerobilia, improved visualization and precision were key contributors to the technique’s success.

**Conclusion:**

Angio-CT may improve procedural precision and safety when aerobilia is present.The integration of CT and angiography within a single system offers a reliable solution for challenging biliary interventions. Larger studies are needed to confirm these findings.

## Introduction

Percutaneous transhepatic biliary drainage (PTBD) is a critical intervention for diagnosing and managing biliary pathologies, such as obstructions, leaks, or infections [1]. Precision during biliary puncture is paramount to avoid complications like vascular injury or bile peritonitis [2, 3]. Conventional fluoroscopy may be insufficient in complex cases, particularly when anatomical landmarks are unclear [3, 4]. Especially in patients with biliary leakage, the non-dilated bile ducts can be very small and difficult to puncture in a conventional way [5, 6]. Usually, an increased amount of punctures is required to successfully gain access to the biliary system increasing the risk for complications [1, 5]. Hybrid angio-CT systems represent an emerging interventional technology that integrates CT and angiography within a single suite, enabling high-resolution cross-sectional imaging and immediate transition to fluoroscopy without patient repositioning [7–10]. These characteristics make the modality particularly attractive for percutaneous transhepatic biliary drainage, where duct visualization and stable instrument guidance are critical for procedural success. It is possible to place a needle using a conventional CT and then transfer the patient to an angiography suite to complete the procedure. This however has high risk of needle or wire dislocation during the transport or transition between beds and intervention tables.

Aerobilia, a condition characterized by the presence of air within the bile ducts, frequently occurs in patients with biliodigestive anastomosis. This condition naturally enhances biliary anatomy on CT, providing a distinct advantage in guiding PTBD procedures. This technical note explores the efficacy and safety of using hybrid angio-CT in ten patients with bile duct leakage, aerobilia, and the need for external biliary drainage.

## Materials and methods

### Patient selection

**Fig. 1:**
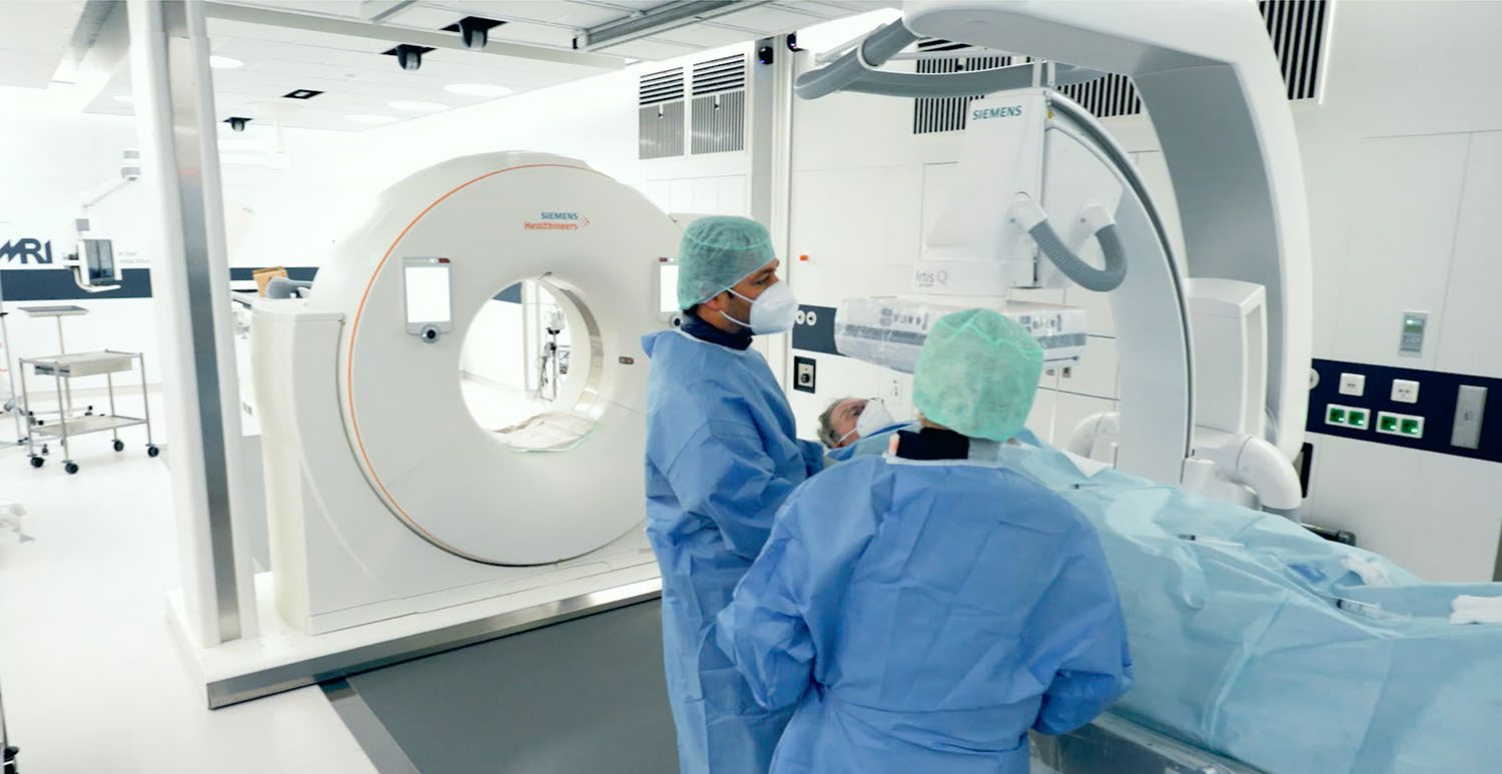
illustrates the setup of the angio CT at our facility

Ten patients with clinically suspected bile duct leakage were included in this analysis. All had undergone previous surgery with creation of a biliodigestive anastomosis for different reasons including malignant and non-malignant pathologies (Fig. 1).

### Procedure

All interventions were performed on a Siemens Nexaris hybrid angio-CT platform with a shared patient table, enabling acquisition of volumetric CT datasets and real-time fluoroscopy within one operational field. The system permits dynamic switching between imaging modes within seconds without altering patient position, thereby securing needle trajectory and guidewire stability throughout the intervention.

**Fig. 2:**
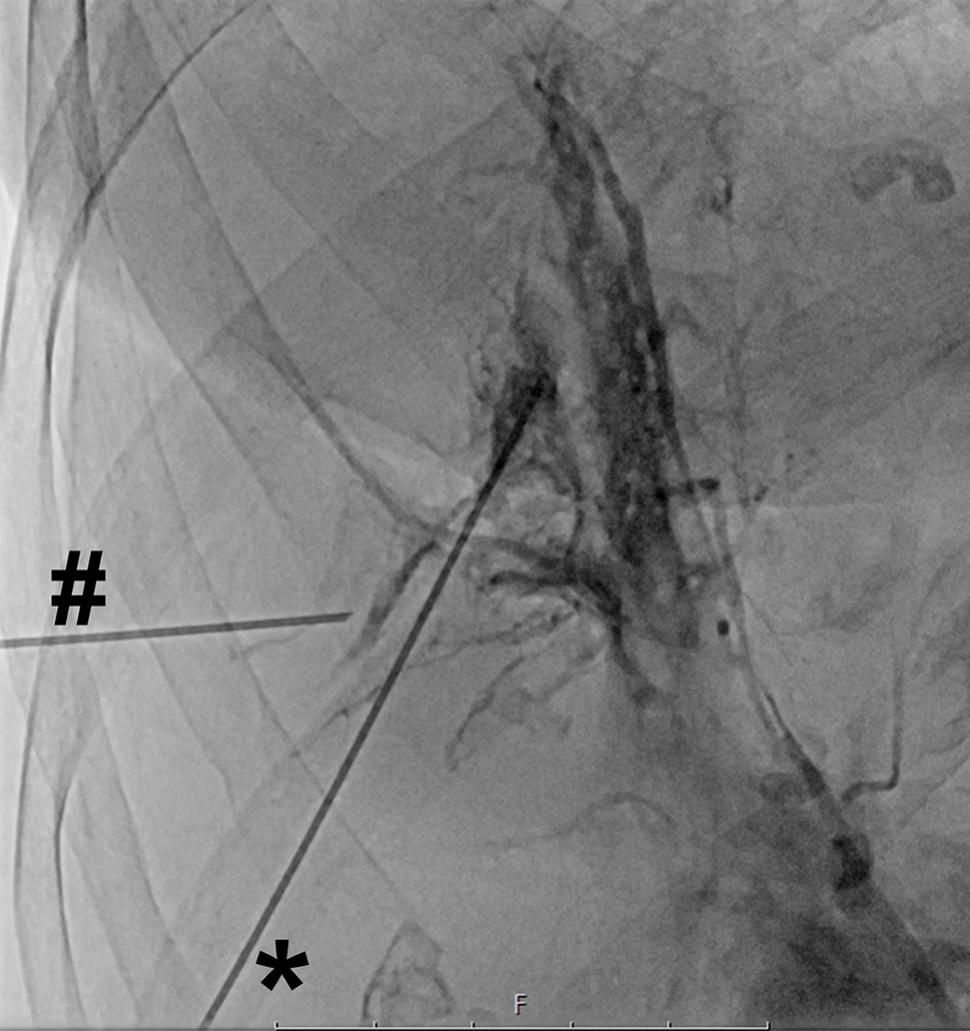
illustrates a complex and ultimately unsuccessful attempt at placing a percutaneous transhepatic cholangial drainage using a conventional approach. The bile ducts are non-dilated and approximately the size of the 22G Chiba needles. The diffuse distribution of contrast media reflects numerous puncture attempts. Additionally, a second 22G Chiba needle was inserted. Through one needle (marked with *), the bile ducts were successfully opacified, but probing them proved unfeasible from this position. To address this limitation, a second needle (marked with #) was used to puncture a bile duct while the biliary system was continuously filled with contrast agent via the first needle (*). This approach compensated for the loss of biliary contrast caused by bleeding into the biliary system from repeated puncture attempts and biliary leakage with high flow

**Fig. 3:**
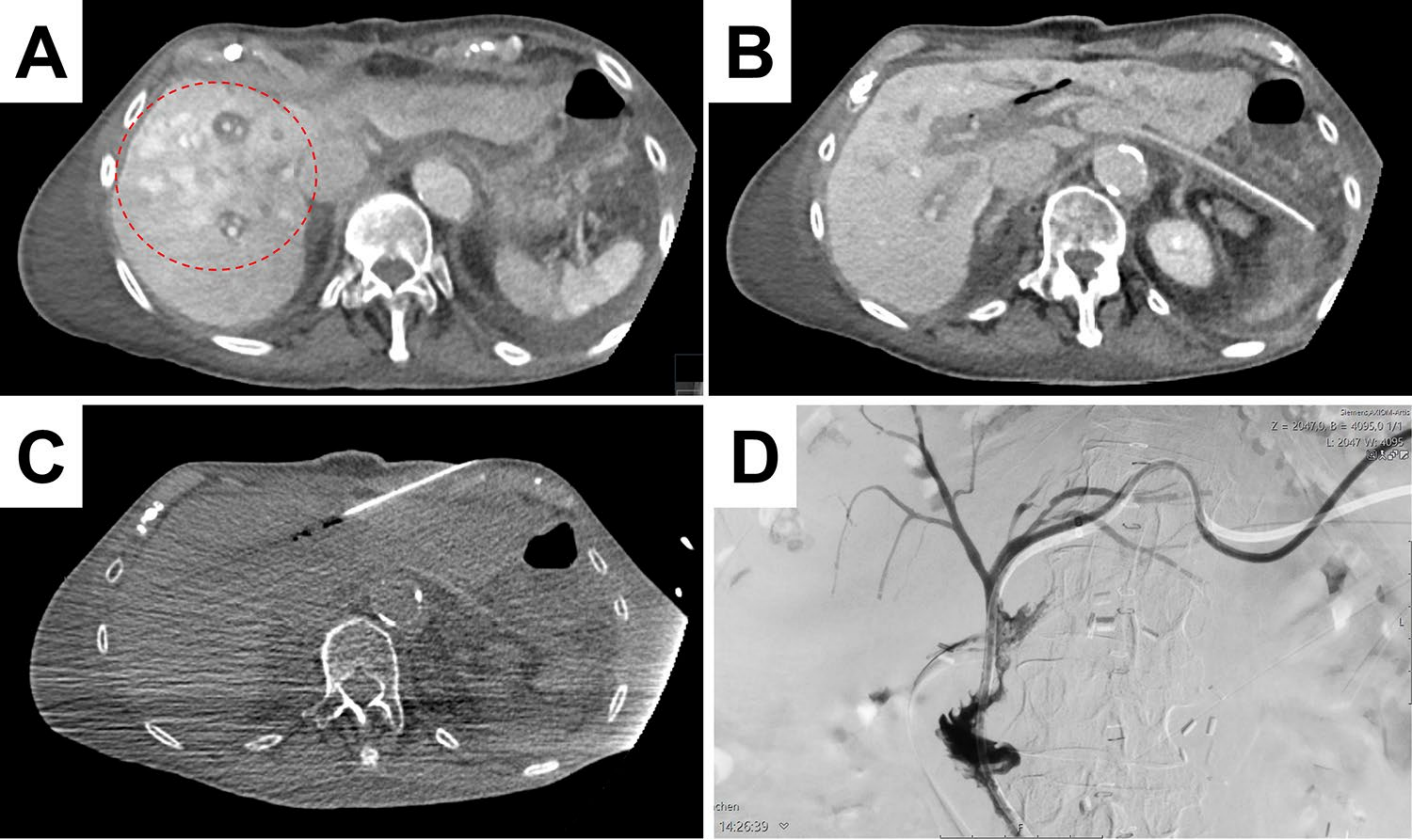
demonstrates the successful placement of a percutaneous transhepatic biliary drainage (PTBD) in the patient from Fig. 2 using the angio CT in hybrid mode. In panel A and B the planning CT for the intervention is shown; the dashed circle(A) highlights an area of focal hyperaemia caused by the numerous puncture attempts performed the previous day. Panel B depicts an optimal left bile duct exhibiting aerobilia and presenting a favorable angle for puncture from the patient’s left side. Panel C shows a CT fluoroscopy image where the needle is being advanced into the bile duct containing aerobilia. Following this, a guidewire was inserted, and a modality switch was performed to conventional angiography. Panel D presents a conventional digital subtraction angiography (DSA) image for confirmation, showing leakage at the anastomosis with drainage into the easy flow drainage system and the correct positioning of the PTBD

Pre-procedural planning involved a non-contrast CT scan for duct mapping. Aerobilia, present due to biliodigestive anastomosis, served as an intrinsic negative contrast agent, sharply outlining non-dilated ducts through air–tissue attenuation gradients. Ducts ≥ 1.5–2 mm were generally visualizable, whereas smaller ducts required sequential dynamic CT fluoroscopy to identify transient air movement. Left-sided ducts were favored based on shorter in-line needle trajectories, anterior orientation, and a higher likelihood of air-filled lumina due to reflux pneumobilia. The skin entry point was selected to optimize a straight-line path to the segment II/III ducts while minimizing traversal of vascular parenchyma (Figs. 2, 3).

CT-fluoroscopy was used for controlled needle advancement. A 22G Chiba needle (180–200 mm length) was advanced under continuous imaging. Needle advancement continued until the tip intersected the targeted aerobilia-filled structure. Intraluminal entry was confirmed based on air reflux at the needle hub or visualization of the needle tip centered within aerated ducts on axial and multiplanar reconstructions.

Once duct entry was confirmed, a 0.018″ Nitrex guidewire was advanced carefully under fluoroscopy. After placement of the 0.018″ wire, CT-fluoroscopy was repeated at a more distal biliary segment to confirm that the wire remained fully intraductal. The system was then switched to conventional fluoroscopy, maintaining the patient and needle-wire assembly completely immobile to avoid tract loss.

A DilPlus^®^ introducer allowed upsizing to a 0.035″ Amplatz wire, which was advanced through the biliary system into the jejunum to achieve a stable track. Over the Amplatz wire, an internal–external PTBD catheter (8–10 F depending on duct calibre) was advanced. Catheter sideholes were positioned proximal and distal to the leak to ensure dual drainage, confirmed using fluoroscopic contrast injection. Flow dynamics, extravasation control, and jejunal runoff were recorded in consecutive digital subtraction angiographies.

## Results

### Visualization and access

Aerobilia provided excellent delineation of the biliary system, particularly in the left-sided ducts and is common in patients with biliodigestive anastomosis. The combined imaging approach facilitated accurate needle placement and reduced the number of puncture attempts to one to three puncture attempts per patient. One patient had undergone conventional but technically unsuccessful PTBD-procedure before the hybrid intervention. Therefore, the technique was developed and was successfully conducted in this patient the next day.

### Procedural success

All ten patients achieved successful biliary access and drainage. The ability to switch seamlessly between CT and fluoroscopy enhanced precision and efficiency. During the evaluation phase one additional patient was supposed to undergo the new procedure but had no signs of aerobilia with collapsed bile ducts in the planning CT. As a consequence, CT guided puncture was not attempted, and the patient underwent successful conventional PTBD procedure; this patient is not included in this presented analysis.

### Safety

No major complications, such as vascular injury, bile peritonitis, or organ damage, were observed. Minor post-procedural pain was managed conservatively.

### Efficiency

The mean procedure time was 25 min (range: 15–35 min). The integration of CT and fluoroscopy in a single system reduced the need for additional imaging modalities and streamlined workflow.

## Discussion

### Advantages of hybrid angio-CT

The integration of CT and fluoroscopy within a single interventional environment provides several important advantages compared to conventional PTBD workflows. Aerobilia as basic requirement acts as an intrinsic negative contrast medium and markedly enhances visualization of even non-dilated bile ducts. This effect is particularly pronounced in left-sided ducts, which are more anteriorly positioned and therefore more readily accessible during image-guided intervention. Improved duct conspicuity allows the operator to identify small-caliber targets that would otherwise require multiple puncture attempts under fluoroscopy alone.

Beyond enhanced visualization, real-time CT fluoroscopy enables continuous monitoring and adjustment of the needle trajectory during hepatic advancement. Deviations can be corrected immediately, reducing the likelihood of extra-ductal placement, parenchymal perforation, or vascular injury. This contributes to a more controlled access pathway, especially in patients with collapsed or narrow duct systems.

A further technical advantage lies in procedural workflow efficiency. Because fluoroscopy and CT operate on a shared gantry and table, modality switching occurs without patient repositioning. This eliminates the logistical break inherent to conventional CT-then-fluoroscopy sequences and prevents guidewire displacement during patient transfer. As a result, the procedure proceeds without interruption, reducing latency between puncture, wire insertion, and catheter deployment while maintaining spatial instrument stability.

### Clinical implications

The use of angio-CT in PTBD represents a significant advancement, particularly for patients with complex biliary anatomy or challenging pathologies [1, 2, 4]. If an angio-CT is available, it should be considered for patients with aerobilia and bile leakage. This approach ensures safe and efficient biliary access while seemingly reducing complications.

### Limitations

This pilot study evaluates the feasibility and safety of the hybrid angio-CT technique. While ultrasound guidance represents an established alternative, it is inherently limited by its dependence on duct visibility, operator skill, and acoustic window quality — factors that are often compromised in non-dilated or post-surgical biliary systems. Availability of hybrid angio-CT systems is limited and broad implementation requires suitable infrastructure. Therefore, this technique currently applies predominantly to centres where such systems are available. Ultrasound-guided access may also be possible when targeting echogenic intraductal air, and angulation of the needle along portal radicles can support duct entry even without CT guidance. In biliary-enteric anastomosis, reflux pneumobilia frequently increases ductal prominence and improves visualisation, independent of hybrid imaging. This study is limited by its small sample size and lack of a control group. Larger, randomized studies are needed to validate the findings and compare outcomes with conventional techniques. This work is a case-series without a comparison cohort; conclusions regarding superiority over conventional PTBD is limited.

## Conclusion

Hybrid angio-CT may be a valuable tool for PTBD in patients with aerobilia and biliodigestive anastomosis. The technique enhances procedural safety, accuracy, and efficiency, providing a reliable alternative to traditional methods. Further research is warranted to establish its role in broader clinical applications.

## Data Availability

The data file is available on reasonable request from the corresponding author.
